# The role of CREG1 in megakaryocyte maturation and thrombocytopoiesis

**DOI:** 10.7150/ijbs.78660

**Published:** 2023-07-09

**Authors:** HaiXu Song, Jiayin Li, Chengfei Peng, Dan Liu, Zhu Mei, Zheming Yang, Xiaoxiang Tian, Xiaolin Zhang, Quanmin Jing, Chenghui Yan, Yaling Han

**Affiliations:** 1 National Key Laboratory of Frigid Zone Cardiovascular Disease, Cardiovascular Research Institute and Department of Cardiology, General Hospital of Northern Theater Command, Shenyang, China.; 2 Northeastern University, Shenyang, China.

**Keywords:** CREG1, megakaryocyte differentiation, thrombopoiesis, MEK-ERK1/2 pathway

## Abstract

Abnormal megakaryocyte maturation and platelet production lead to platelet-related diseases and impact the dynamic balance between hemostasis and bleeding. Cellular repressor of E1A-stimulated gene 1 (CREG1) is a glycoprotein that promotes tissue differentiation. However, its role in megakaryocytes remains unclear. In this study, we found that CREG1 protein is expressed in platelets and megakaryocytes and was decreased in the platelets of patients with thrombocytopenia. A cytosine arabinoside-induced thrombocytopenia mouse model was established, and the mRNA and protein expression levels of CREG1 were found to be reduced in megakaryocytes. We established megakaryocyte/platelet conditional knockout (*Creg1^pf4-cre^*) and transgenic mice (tg-*Creg1*). Compared to* Creg1^fl/fl^* mice, *Creg1^pf4-cre^* mice exhibited thrombocytopenia, which was mainly caused by inefficient bone marrow (BM) thrombocytopoiesis, but not by apoptosis of circulating platelets. Cultured *Creg1^pf4-cre^*-megakaryocytes exhibited impairment of the actin cytoskeleton, with less filamentous actin, significantly fewer proplatelets, and lower ploidy. CREG1 directly interacts with MEK1/2 and promotes MEK1/2 phosphorylation. Thus, our study uncovered the role of CREG1 in the regulation of megakaryocyte maturation and thrombopoiesis, and it provides a possible theoretical basis for the prevention and treatment of thrombocytopenia.

## Introduction

Platelets are an important part of circulating blood in the body, the disorder of bleeding and hemostasis is a serious threat to human health^1^-^2^. Megakaryocytes are precursors of platelets that mainly exist in the bone marrow. They are generated by specific differentiation of hematopoietic stem cells, undergoing a unique process of differentiation known as polyploidy. Polyploid megakaryocytes undergo endomitosis and rapid cytoplasmic expansion, forming a complex demarcation membrane system (DMS) that synthesizes proteins and particles necessary for platelet function^3^. Platelet production is a complex process regulated in multiple stages^4^.

Previous studies have demonstrated that the MEK-ERK1/2 signaling pathway plays a critical role in megakaryocyte differentiation, motility, and proplatelet formation^5^,^6^. Activation of the MEK/ERK1/2 pathway is sufficient to induce an increase in the megakaryocyte commitment marker (GPIb) and promotes a rightward shift of the ploidy. Megakaryocyte development and proplatelet formation are regulated by a variety of transcription factors, cytokines, and protein kinases via the MEK/ERK1/2 pathway and cytoskeleton reorganization. Yang et al^7^. also revealed that serotonin, as a growth factor for hematopoietic stem/progenitor cells, plays an important role in platelet formation by activating the ERK1/2 pathway. However, the mechanism of abnormal megakaryocyte differentiation and platelet biogenesis has not been fully elucidated.

In this study, we aimed to determine the role of CREG1 (cellular repressor of E1A-stimulated genes 1) in megakaryocyte maturation and platelet biogenesis. CREG1 is a low-molecular-weight secreted glycoprotein composed of 220 amino acids^8^, that is widely expressed in mature tissue cells and that has ability to maintain tissue and cell maturity^9^. Studies have shown that CREG1 expression is low in undifferentiated embryonic stem cells and is rapidly upregulated upon differentiation. In addition, CREG1 overexpression increases retinoic acid-induced embryonal carcinoma cell differentiation into a neuronal lineage. Similarly, it has been shown that CREG1 promotes embryonic stem cell differentiation into cardiomyogenic cells^10^. Moreover, CREG1 induces vascular smooth muscle cell differentiation in embryonic stem cells^11^. These studies on embryonic stem cells indicate that CREG1 could be a homeostatic regulator that induces cell differentiation. We found that CREG1 is highly expressed in megakaryocytes and platelets; however, the role of CREG1 in megakaryocyte maturation from stem cells and platelet production remains to be elucidated.

In the present study, we show that megakaryocyte/platelet-specific *Creg1*-deficient mice (*Creg1^pf4-cre^* mice) develop thrombocytopenia. Mechanistic studies revealed that *Creg1* deficiency results in inactivation of the MEK-ERK1/2 pathway. Our study demonstrates the important role of CREG1 in megakaryocyte differentiation and platelet production and suggests that CREG1 may be a novel target for the treatment of thrombocytopenia.

## Materials and methods

### Patients information

A total of 10 patients treated with chemotherapy drugs and 10 control subjects (Supplementary Table 1) were enrolled in this study between 2017 and 2019. The study was approved by the Ethics Committee of the General Hospital of Shenyang Northern Theater Command of China (K2017-16). Informed consent was obtained from all participants in the study. The reference range of platelet counts in normal humans at our center is 125-350 × 10^9^/L.

### Mice

*Creg1*-floxed mice (*Creg1^fl/fl^*), megakaryocyte-platelet-specific platelet factor 4-*Cre* mice (*Pf4*-Cre), and *Creg1* transgenic mice (tg-*Creg1*) were generated by the Research Center of Southern Model Organisms (Shanghai, China). *Creg1^fl/fl^pf4*-Cre^+^ (*Creg1^pf4-cre^*), *Creg1^fl/fl^pf4*-Cre^-^ (*Creg1^fl/fl^*), and tg-*Creg1* mice were bred and genotyped. All mice were fed and placed in a 12:12 h light:dark cycle system using an automated light-switching system and temperature-controlled conditions at 22°C. As described previously^12^-^14^, the mice were intraperitoneally injected with cytosine arabinoside (9PD5221, Actavis, Italy) for 2 days (200 mg/kg/day) or 50 mg/kg/day for 3 days, the control group was injected with saline. Because of its obvious inhibitory effect on the growth of bone marrow megakaryocytes, cytosine arabinoside (Ara-c) was used to cause secondary thrombocytopenia in mice. Blood samples were collected after 5 and 10 days of Ara-c treatment and subjected to determination of WBC and RBC counts, the HGB content, and the MPV of platelets in the peripheral blood using standard clinical blood chemistry procedures. All experiments were approved by the Animal Ethics Committee of the General Hospital of Northern Theater Command and were conducted in accordance with existing guidelines on the care and use of laboratory animals.

### Antibodies and reagents

TPO (Cat. no. 1624) was purchased from Tocris (Minneapolis, MN, USA). JYBL-1 was from Sigma-Aldrich (Solarbio Life Sciences), and TRIzol^®^ reagent was purchased from Selleckchem (Invitrogen). SYBR™ Premix Ex Taq II was purchased from Thermo Fisher Scientific (Waltham USA, MA). CREG1 was purchased from Sigma-Aldrich (09015-1B7) and HUABIO (ER61836). The DyLight™ 488 conjugation kit, and anti-CD42a, anti-CD42b, and anti-CD41 antibodies, were purchased from Abcam (Cambridge, USA). Anti-GAPDH, anti-cleaved caspase-3, anti-phospho-MEK1/2, anti-MEK1/2, anti-phospho-ERK1/2 (Thr202/Tyr204), anti-ERK1/2, anti-p38, anti-phospho-p38 (Thr180/Tyr182), anti-phospho-PAK1/2 (Ser209), anti-phospho-LIMK1, anti-phospho-cofilin, anti-total PAK, anti-cofilin, and anti-total LIMK1 antibodies were obtained from Cell Signaling Technology (Danvers, MA, USA). PE-CD41 was obtained from BD Biosciences, and FITC-CD42b was obtained from Emfret Analytics. U0126 and propidium iodide were obtained from Sangon Biotech (Shanghai, China).

### Platelets preparation and Annexin V staining

Blood was collected from the abdominal aorta of mice, and washed platelets were prepared by gradient centrifugation, as described previously^15^. The washed platelets were resuspended in annexin V binding buffer and incubated with annexin V-FITC for 30 min. The samples were analyzed using a flow cytometer (BD Biosciences) and the data were analyzed using FlowJo™ software (FlowJo, Ashland, OR, USA).

### Detection of platelet lifespan in mice

DyLight™ 488 fluorescently labeled rabbit anti-GPIX (CD42a) antibody was injected into mice by tail vein injection, and blood was collected before and after injection at different time points, as described previously^15^. Cytometry was used to determine the lifespan of platelets in different groups of mice, and the platelet clearance rate was determined.

### Detection of platelet production in mice

Rabbit anti-GPIbα (CD42b) antibody was injected into mice by tail vein injection. The number of platelets was measured before and after the injection, and a platelet production curve was generated from the data.

### TPO intervention

Thrombopoietin (TPO) was injected into mice by tail vein injection, and the number of platelets was measured before and on the 4^th^ day after injection.

### Megakaryocyte differentiation, proplatelet production, and polyploid detection

As described previously^15^, bone marrow was obtained by flushing the femur with phosphate-buffered saline (PBS), and fetal livers were isolated from 13.5~17-day-old mouse embryos. Human stem cells were obtained from Procell Life Science Technology Co., Ltd. (CP-H185). The cells were then suspended in 10 mL DMEM containing 10% FBS, and 10 ng/mL interleukin-3 (IL-3) along with 15 IU/mL recombinant human TPO were added *in vitro* for 4~5 d*.* Megakaryocytes were obtained by bovine serum albumin (BSA) gradient centrifugation.

To assay megakaryocyte proplatelet formation (PPF), glass slides were coated with a fibrinogen (50 μg/mL) solution overnight at 4 °C. TPO (15 IU/mL) was added to the culture medium of megakaryocytes after resuspension, and cell morphology was observed using a microscope in a 5% CO_2_ incubator at 37 °C for 14 h. The megakaryocytes were then fixed with 4% paraformaldehyde for 30 min, stained with α-tubulin and Alexa Fluor 488-conjugated anti-rabbit antibody, and analyzed by confocal microscopy. Images were processed using ImageJ software.

Flow cytometry was used for the megakaryocyte polyploidy analysis. Megakaryocytes were stained with FITC-CD41 (BD Biosciences) and fixed in 70% cold alcohol overnight at 4°C. After washing, megakaryocytes were stained with 20 μL propidium iodide (A601112-0020, Sangon Biotech, Shanghai, China) in PBS containing 0.1% Triton-X 100 and 100 U/mL RNase (Sangon Biotech). DNA ploidy was analyzed using FlowJo™ 7.6.1 software (FlowJo).

### Cell culture, small interfering RNA and Plasmid construction

Dami (megakaryocyte cell line) cells were cultured in a 10% FBS 1640 DMEM medium and HEK293T cells were cultured in a 10% FBS medium (Gibco, Thermo Fisher Scientific) in 5% CO_2_ at 37 °C. Small interfering RNAs (siRNAs) of *Creg1* (RiboBio) and the negative control (si*Scramble*) were transfected into cells using Lipofectamine™ 2000 reagent (Thermo Fisher Scientific). Dami cells were treated with 100 nM PMA (A606759, Sangon Biotech) for 4 days to induce megakaryocyte differentiation. Dami cells were treated with saline or 1 µM U0126 for 1 h, and the effect of U0126 on PMA-induced differentiation was examined for 72 h.

### Real-time polymerase chain reaction (PCR)

Total RNA was extracted from megakaryocytes and Dami cells using the TRIzol^®^ reagent (Invitrogen Life Technologies, USA). cDNA was synthesized using the SuperScript™ III First-Strand Kit (18080400, Thermo Fisher Scientific), as described previously. Real-time PCR was performed using an ABI 7300 PCR System (Thermo Fisher Scientific). Gene expression levels were normalized to *GAPDH* and analyzed using the 2∆∆Ct method. The primer sequences (5' and 3') used were as follows: m*Gapdh*-F: AGGTCGGTGTGAACGGATTTG, m*Gapdh-*R: TGTAGACCATGTAGTTGAGGTCA; m*Creg1-F*: GTGGCACTACTGGTGTCGC, m*Creg1*-R: CGCGCACCTCCTTTATTGTG;* hGAPDH*-F: ACAACTTTGGTATCGTGGAAGG,* hGAPDH*-R: GCCATCACGCCACAGTTTC; h*CD41*-F: GATGAGACCCGAAATGTAGGC; h*CD41*-R: GTCTTTTCTAGGACGTTCCAGTG; h*CREG1-F*: GACTTTGGCACAGACCAACTT, h*CREG1*-R: CAGGGTGTCGAATGAATAACGA.

### Western blot analysis

Platelets, megakaryocytes, and Dami cells were lysed using RIPA buffer (Thermo Fisher Scientific) for 30 min on the ice and then centrifuged at 12,000 × g for 10 min at 4°C. Equal amounts of sample were separated by SDS-PAGE (80~120 V for 1.5 h) and then transferred onto polyvinylidene difluoride membranes. The membranes were incubated with antibodies against GAPDH (5174S, Cell Signaling Technology), integrin alpha-IIb (CD41; ab33661, Abcam), p44/42 mitogen-activated protein kinase (MAPK) extracellular signal-regulated kinase 1/2 (ERK1/2; 4695S, Cell Signaling Technology), phospho-p44/42 MAPK ERK1/2 (4370S, Cell Signaling Technology), JNK (9252T, Cell Signaling Technology), p-JNK(9255S, Cell Signaling Technology), p-p38(9211S, Cell Signaling Technology), and p38 (9212S, Cell Signaling Technology) at 4 °C overnight after blocking of the membranes with 5% non-fat dry milk. All antibodies were diluted to 1:1000. The membranes were then incubated with goat anti-rabbit or anti-mouse antibodies (1:5000, Thermo Fisher Scientific) for 2 h at 25 °C. Protein bands were quantified using ImageJ software.

### Immunohistochemical staining of mouse femur and spleen

The mice were anesthetized by intraperitoneal injection of 4% chloral hydrate, and the femurs were immersed in 4% paraformaldehyde for 48 h. The femur tissue was removed and rinsed three times for approximately 20 min with PBS, followed by three times (20 min each) with distilled water. The femurs required further decalcification. Each femur was transferred to a 50 mL centrifuge tube containing 20 mL of JYBL-1 decalcification solution. The decalcification time was approximately 24~36 hours, and the decalcification speed was increased at 37 °C. When the femur tissue became soft, the decalcification was stopped and the tissue repeatedly cleaned with distilled water. The femur and spleen tissues were removed and placed in a tissue dehydration and embedding machine for dehydration, transparency, and paraffin embedding, and then stained, as described previously^3^.

### Wright-Giemsa staining

As described previously^15^, cytospin slides of megakaryocytes were stained using a Wright-Giemsa Stain Kit (BA-4017, Baso). Images were obtained using a fluorescence microscope (Carl Zeiss). The data were analyzed using ZEN software (Carl Zeiss).

### Transmission electron microscopy (TEM)

Isolated megakaryocytes and platelets were fixed with 2.5% glutaraldehyde buffer, as described previously^16^. The fixed megakaryocytes and platelets were processed for TEM, sectioned, and imaged.

### Immunoprecipitation (IP)

293T cells and primary megakaryocytes transfected with pcDNA3.1-CREG1-Flag (WZ Biosciences Inc.) and pcDNA3.1-MEK1/2-GFP (GENEWIZ) were collected, homogenized, and lysed with IP lysis buffer (Thermo Scientific) for 30 min on ice. The lysates were centrifuged at 12,000 × g for 10~15 min and the supernatants were incubated with antibody-conjugated beads (Thermo Fisher Scientific, WF322336) and mixed overnight by rotation at 4°C. The next day, the supernatants were discarded, and the beads were washed three times with 800 μL PBS. Western blot of the samples was then performed, as described previously.

### Statistical analysis

All data are expressed as means ± standard error (SEM). All data were analyzed using SPSS (version 13.0; SPSS, Chicago, IL, USA) and GraphPad Prism 8.0 statistical software. The Shapiro-Wilk test was used for normality and Levene's test was used for homogeneity of variance. When the data conformed to both normal distribution and homogeneity of variance, differences between the two groups were compared using paired or unpaired t-tests, one-way analysis of variance (ANOVA), and repeated measures of variance. When the data did not conform to a normal distribution, the method used was a non-parametric test. P < 0.05 was considered statistically significant.

## Results

### Reduced CREG1 expression in the platelets of thrombocytopenia patients and megakaryocytes of mice with thrombocytopenia induced by cytosine arabinoside

**Figure 1:**
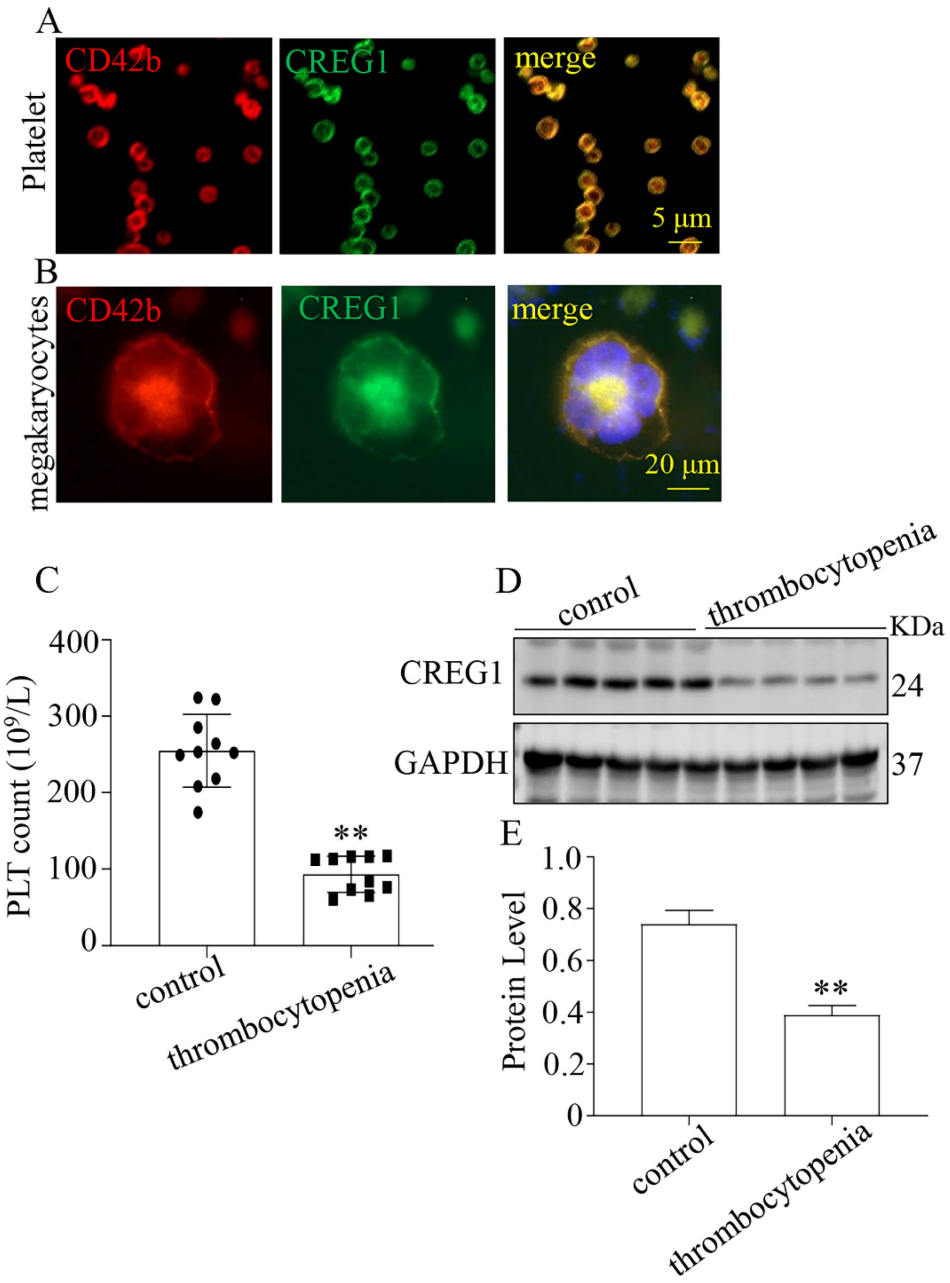
** CREG1 protein was decreased in the platelets of thrombocytopenia patients.** (A-B) Immunofluorescence staining of CREG1 in platelets and megakaryocytes of human (n=5). (C) platelet count (n=10). (D-E) CREG1 protein levels were determined by western blot in thrombocytopenia patients and controls (n=3). Values are means ± SEM. ^**^P < 0.01 versus control.

**Figure 2:**
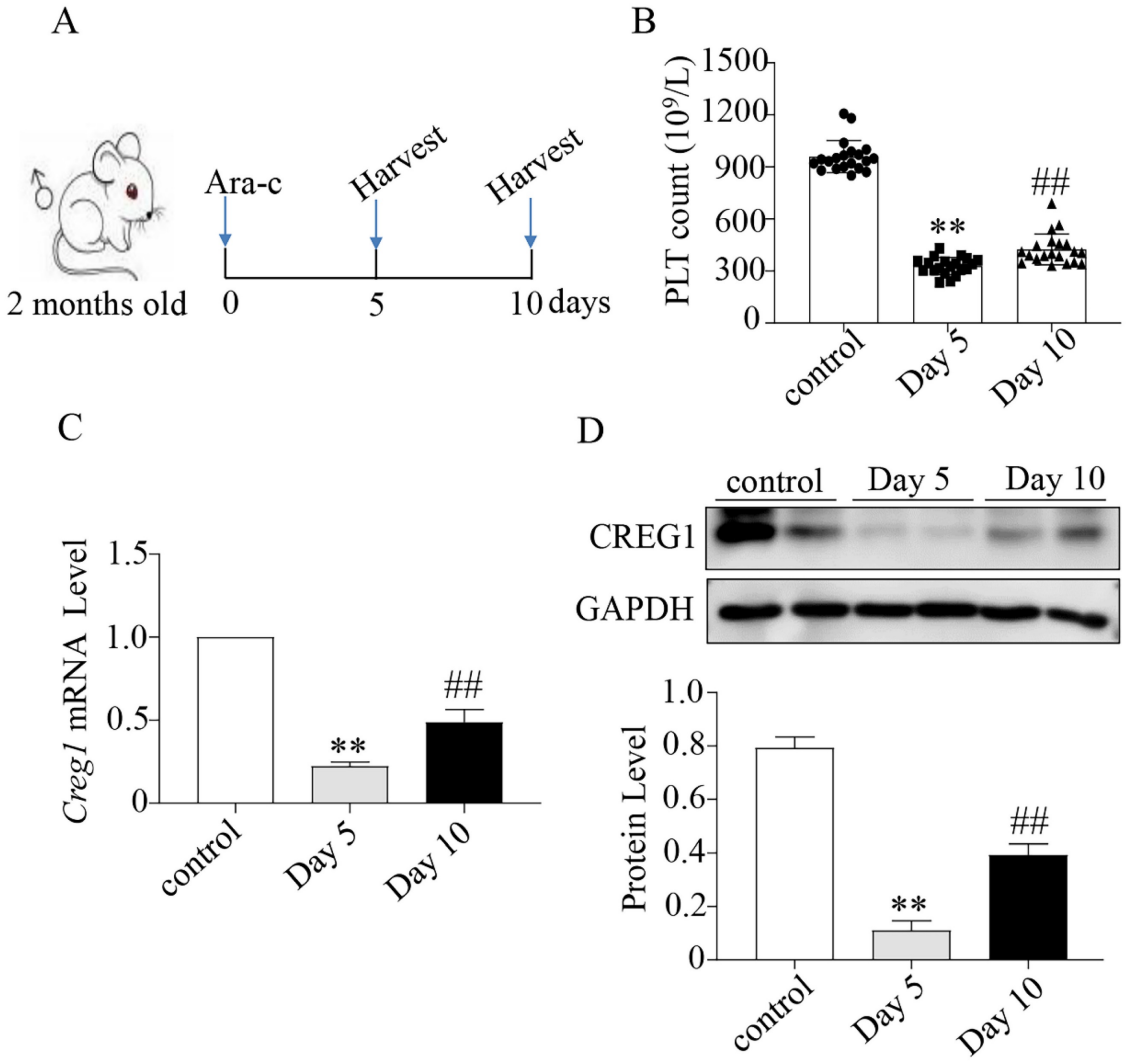
** Expression of CREG1 was decreased in megakaryocytes of mice treated with cytosine arabinoside.** (A) Mice treated intraperitoneally with Ara-c. (B) PLT count in the peripheral blood. (C) Expression of *Creg1* mRNA by real-time PCR after treatment with cytosine arabinoside for 5 and 10 days. (D) CREG1 protein was analyzed by western blot in megakaryocytes. Values are means ± SEM. n=20 mice/group. **P < 0.01 versus control, ^##^*P* < 0.01 versus Day 5. Ara-c: cytosine arabinoside.

We verified that CREG1 is expressed in platelets and megakaryocytes of human (Figures 1A-B). CREG1 protein levels were measured in the platelets of 10 thrombocytopenia patients and 10 control subjects (Supplementary Table 1), and markedly decreased in platelets from patients (Figures 1C-E). Ara-c was administered to mice to establish a thrombocytopenia model^12^-^14^. As shown in Supplementary Figures 1A-D, the WBC and RBC counts and hemoglobin (HGB) content of the model group markedly decreased on the 5th day, but there was no statistical significance on the 10^th^ day compared with the control. The platelet count on the 10^th^ day remained significantly decreased, indicating that the thrombocytopenia model was successfully established (Figures 2A-B). CREG1 mRNA and protein expression levels in megakaryocytes of mice induced by Ara-c were decreased (Figures 2C-D).

### CREG1 deficiency caused thrombocytopenia in mice

**Figure 3:**
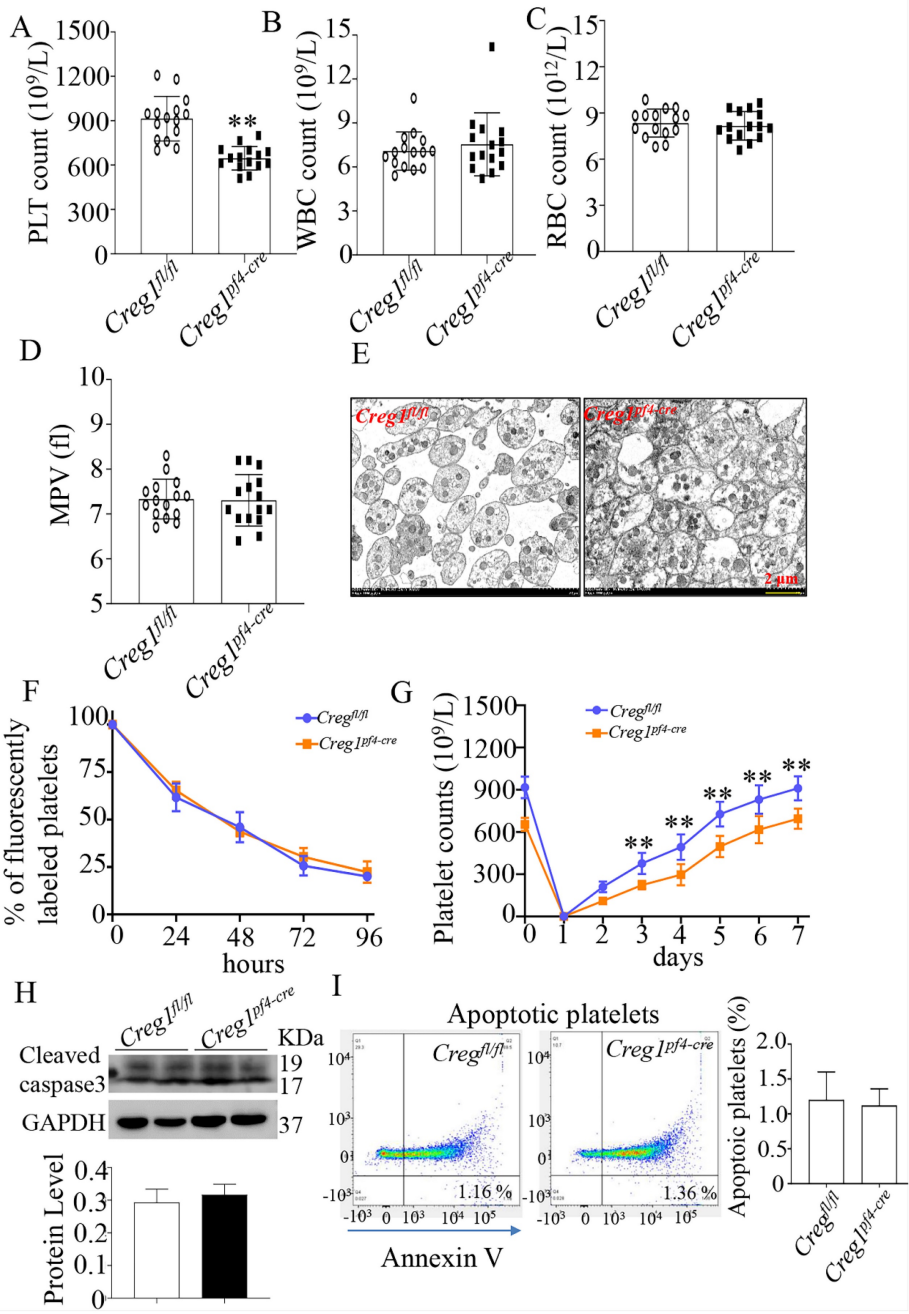
** Lack of CREG1 led to reduced platelet production.** (A-C) PLT, RBC, and WBC counts in the peripheral blood. (D) MPV; (n=15). (E) Transmission electron microscopy (TEM) analysis of platelets; (n=5). (F) Platelet survival was measured after intravenous injection of DyLight™ 488-conjugated anti-GPIbβ Ig derivative (0.1 µg/g body weight) in the tail vein. (G) Platelets were first eliminated by intravenous injection of anti-CD42b (2 mg/g) in the tail vein. (H) Western blot was used to analyze cleaved caspase-3 expression in platelets. (I) Apoptotic platelets were detected by annexin V staining using flow cytometry; (n=3). Values are means ± SEM. **P < 0.01 versus* Creg1^fl/fl^*. MPV: mean platelet volume.

To explore the role of CREG1 in megakaryocyte maturation and platelet production, we generated a megakaryocyte/platelet-conditional *Creg1* knockout mouse (*Creg1^pf4-cre^*). *Creg1*-floxed mice (*Creg1^fl/fl^*) were crossed with a transgenic line expressing *Cre* recombinase under the megakaryocyte/platelet-specific* pf4* promoter (Supplementary Figure 2A). Quantitative real-time PCR and western blot revealed the expression of CREG1 transcripts and protein, respectively (Supplementary Figures 2C-F). As expected, the platelet count of *Creg1^pf4-cre^* mice (646.2 ± 20.55 × 10^9^/L) was lower than that of *Creg1^fl/fl^* mice (913.9 ± 38.89 × 10^9^/L) (Figure 3A). On the other hand, *Creg1^fl/fl^* and *Creg1^pf4-cre^* mice had similar WBC, RBC, and MPV values (Figures 3B-D). Transmission electron microscopy also showed that there was no difference in platelet size between *Creg1^pf4-cre^* and *Creg1^fl/fl^* mice (Figure 3E).

Furthermore, we explored the cause of the thrombocytopenia in *Creg1^pf4-cre^* mice, and we measured the rates of platelet clearance and regeneration. The results showed that *Creg1^pf4-cre^* mice had normal platelet lifespans compared with those of *Creg1^fl/fl^* mice (Figure 3F), based on the proportions of DyLight™ 488 labeled rabbit anti-GPIX platelets at different time points. We also found that CREG1 deficiency significantly impaired thrombopoiesis when anti-CD42b monoclonal antibodies were administered to delete platelets (Figure 3G). Platelets from the peripheral blood were detected by annexin V staining and western blot, and the results indicated that there was no difference in apoptosis between *Creg1^fl/fl^* and* Creg1^pf4-cre^* mice (Figures 3H-I).

### CREG1 deficiency significantly impaired megakaryocyte differentiation and PPF formation

**Figure 4:**
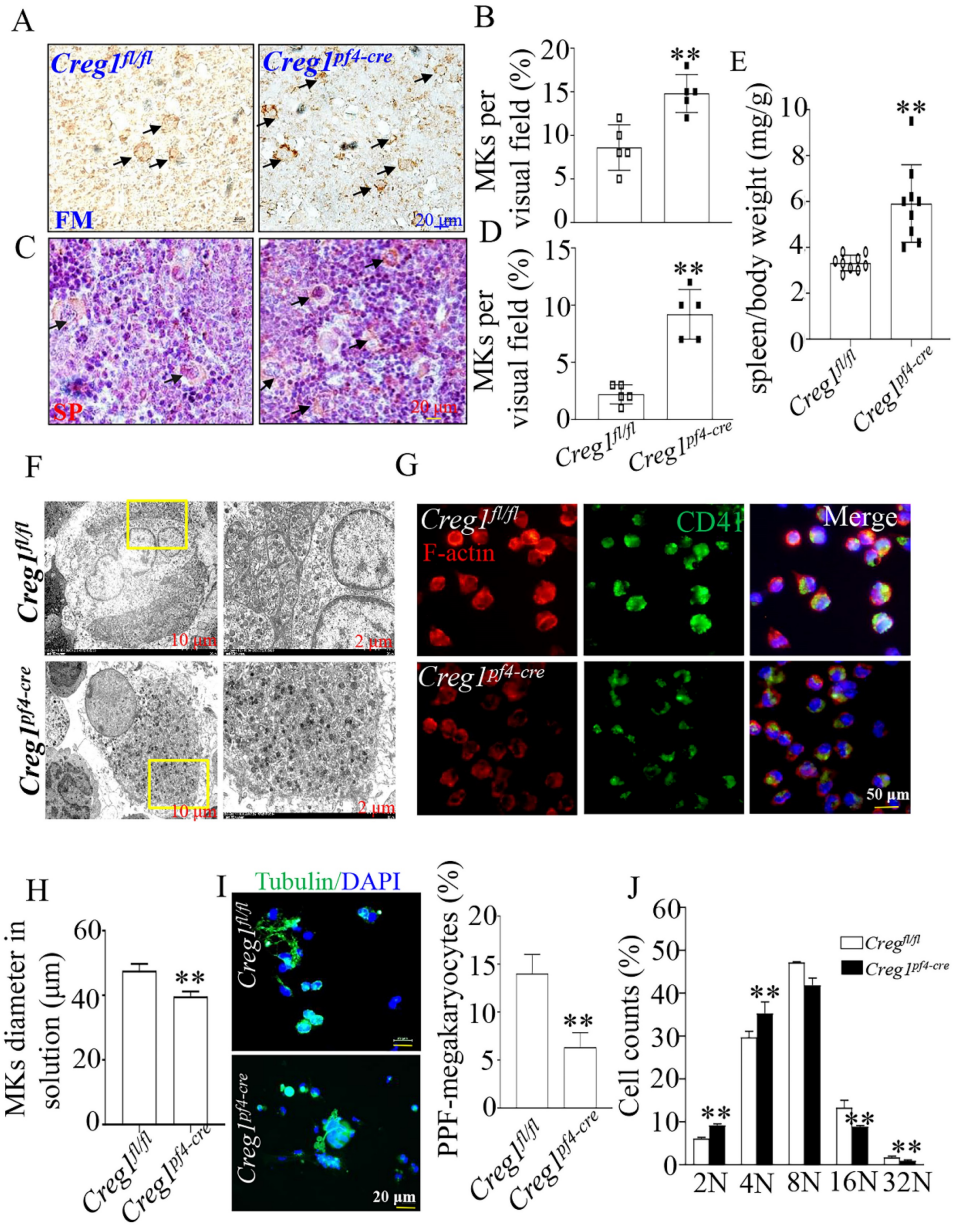

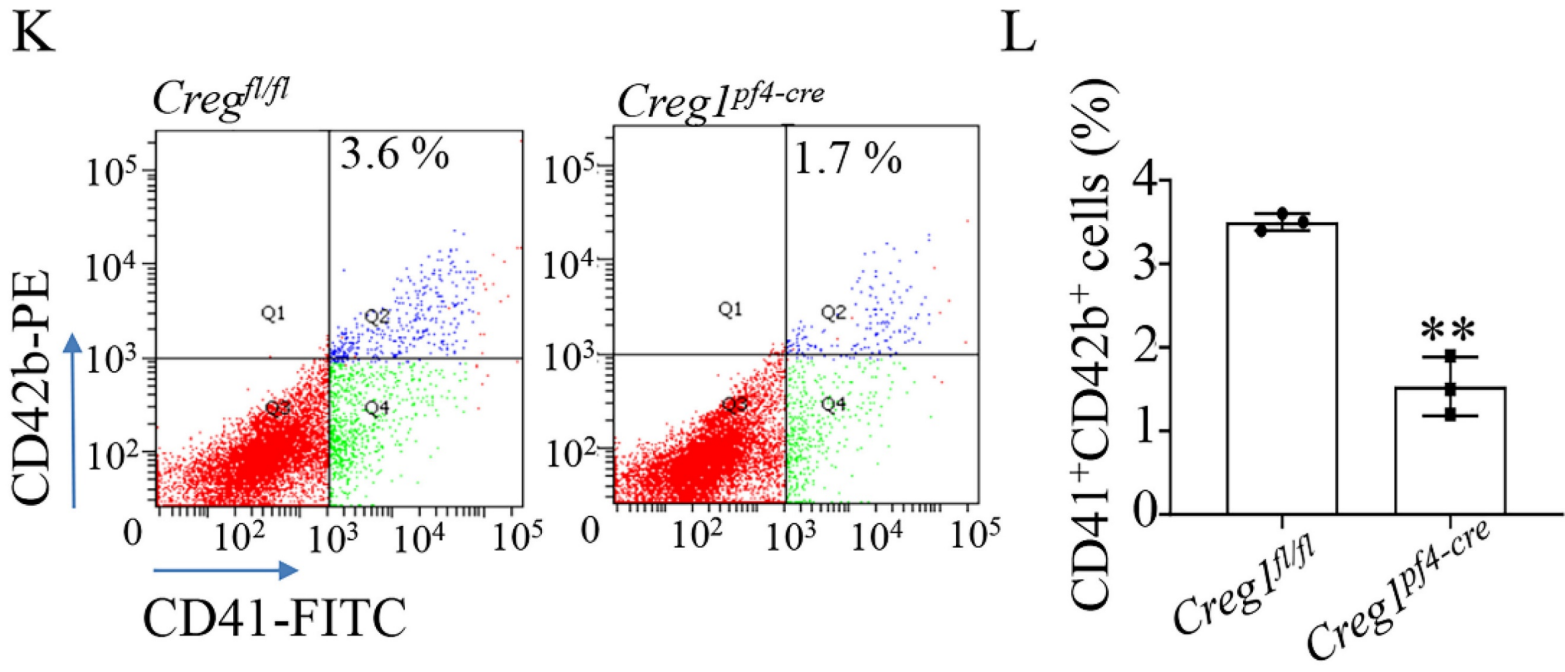
** Impaired megakaryocyte development in *Creg1^pf4-cre^* mice.** (A-D) Immunohistochemical images of CD42b (GPIb) in the femurs and spleens of *Creg1^fl/fl^* and* Creg1^pf4-cre^* mice; (n=5). (E) Spleen/body weight; (n=9). (F) TEM analysis of bone marrow (BM) megakaryocytes. (G-H) Confocal images of BM-derived megakaryocytes cultured *in vitro* for five days and stained for F-actin. (I) Immunofluorescence images of α-tubulin in PPF-megakaryocytes derived from the fetal livers of *Creg1^fl/fl^* and *Creg1^pf4-cre^* mice; (n=5). (J) Percentage of megakaryocytes polyploidy; (n=3). (K-L) Percentage of CD41^+^CD42b^+^ in fetal livers derived from *Creg1^pf4-cre^* and *Creg1^fl/fl^* mice were measured by flow cytometry, (n=3). Values are presented as means ± SEM. **P < 0.01 versus *Creg1^fl/fl^*. MKs: megakaryocytes.

To investigate the origin of the observed impaired thrombopoiesis, megakaryocyte development was assayed by immunohistochemical staining for CD42b in the femurs and spleens of *Creg1^fl/fl^* and* Creg1^pf4-cre^* mice. As shown in Figures 4A-B, the bone marrow (BM) of *Creg1^pf4-cre^* mice exhibited significant megakaryocyte hyperplasia, as reflected by the increased number of CD42b-positive megakaryocytes. The spleen is a known site of platelet production when BM-derived thrombopoiesis is not sufficient^3^. As shown in Figures 4C-E, we found that the number of CD42b-positive megakaryocytes was markedly increased in the spleen sections of *Creg1^pf4-cre^* mice, and *Creg1^pf4-cre^* mice developed splenomegaly compared to *Creg1^fl/fl^* mice. CREG1 deficiency resulted in a decreased platelet count and compensatory increased megakaryocyte numbers in the BM and spleen, indicating that the lack of CREG1 affected platelet production.

Next, we analyzed the morphology and polarization of megakaryocytes in *Creg1^pf4-cre^* mice. Transmission electron microscopy was used to investigate megakaryocyte ultrastructure in the BM. We found that the DMS of megakaryocytes in *Creg1^pf4-cre^* mice had fewer invaginations in the periphery and around the nucleus than those in *Creg1^fl/fl^* mice (Figure 4F). Furthermore, the diameter of cultured megakaryocytes of *Creg1^pf4-cre^* mice was significantly reduced (Figures 4G-H), as observed by immunofluorescence, and previous studies have identified a DMS polarization process that is dependent on actin reorganization^17^,^18^.

Fetal livers were isolated from *Creg1^fl/fl^* and *Creg1^pf4-cre^* embryos and incubated with IL-3 and TPO for five days, followed by BSA gradient separation to obtain megakaryocytes. The results showed that *Creg1^pf4-cre^* megakaryocytes produced fewer proplatelets and were morphologically less differentiated than *Creg1^fl/fl^* megakaryocytes, as determined using immunofluorescence confocal microscopy (Figure 4I). Flow cytometry analysis of DNA ploidy revealed that *Creg1^pf4-cre^* megakaryocytes included more cells delayed in the tetraploid and octaploid phases (4N-8N), but fewer cells in higher ploidy phases (> 16N) (Figure 4J). The percentages of CD41^+^CD42b^+^ megakaryocytes in fetal livers which were derived from *Creg1^pf4-cre^* and *Creg1^fl/fl^* mice and treated with IL-3 and TPO, were also measured by flow cytometry. In the *Creg1^pf4-cre^* mice, 1.7% of the cells were CD41^+^CD42b^+^, whereas in the *Creg1^fl/fl^* mice, 3.6% of the cells were CD41^+^CD42b^+^ (Figures 4K-L). These results suggest that CREG1 is an important regulator of megakaryocyte maturation.

### Administration of TPO did not sufficiently improve thrombocytopenia due to CREG1 deficiency

TPO and C-MPL receptors are classic signaling pathways that promote megakaryocyte differentiation and platelet formation^19^, some studies have also found that decreased platelet production may not be associated with the TPO pathway^7^,^20^. To clarify the relationship between CREG1 loss, leading to reduced platelet formation, and the TPO signaling pathway, we used a TPO ELISA kit to detect serum TPO concentrations in *Creg1^fl/fl^* and *Creg1^pf4-cre^* mice. The results showed that there was not a significant difference in the serum TPO concentrations between the two groups (Supplementary Figure 3A). Western blot was used to detect the expression of the TPO receptor C-MPL protein in megakaryocytes, and no significant difference was observed between the two groups (Supplementary Figures 3B-C). In addition, to determine the effect of TPO on platelet formation in *Creg1^pf4-cre^* mice, the baseline platelet count of the mice before and four days after continuous injection of recombinant TPO via tail vein injection was determined. The platelet counts of *Creg1^fl/fl^* mice significantly increased after TPO injection compared with those before the injection (1136.7 ± 49.2 × 10^9^/L vs. 882.6 ± 36.1 × 10^9^/L, respectively). While TPO administration failed to sufficiently elevate platelet counts of *Creg1^pf4-cre^* mice to prevent the thrombocytopenia, the number of platelets in *Creg1^pf4-cre^* mice compared with that before TPO injection (821.3 ± 32.8 × 10^9^/L vs. 633.3 ± 26.2×10^9^/L, respectively) (Supplementary Figure 3D). These results suggest that *Creg1* deletion may attenuate the role of TPO signaling pathway in platelet production.

### CREG1 overexpression increased the platelet counts of mice treated with cytosine arabinoside *in vivo*

**Figure 5:**
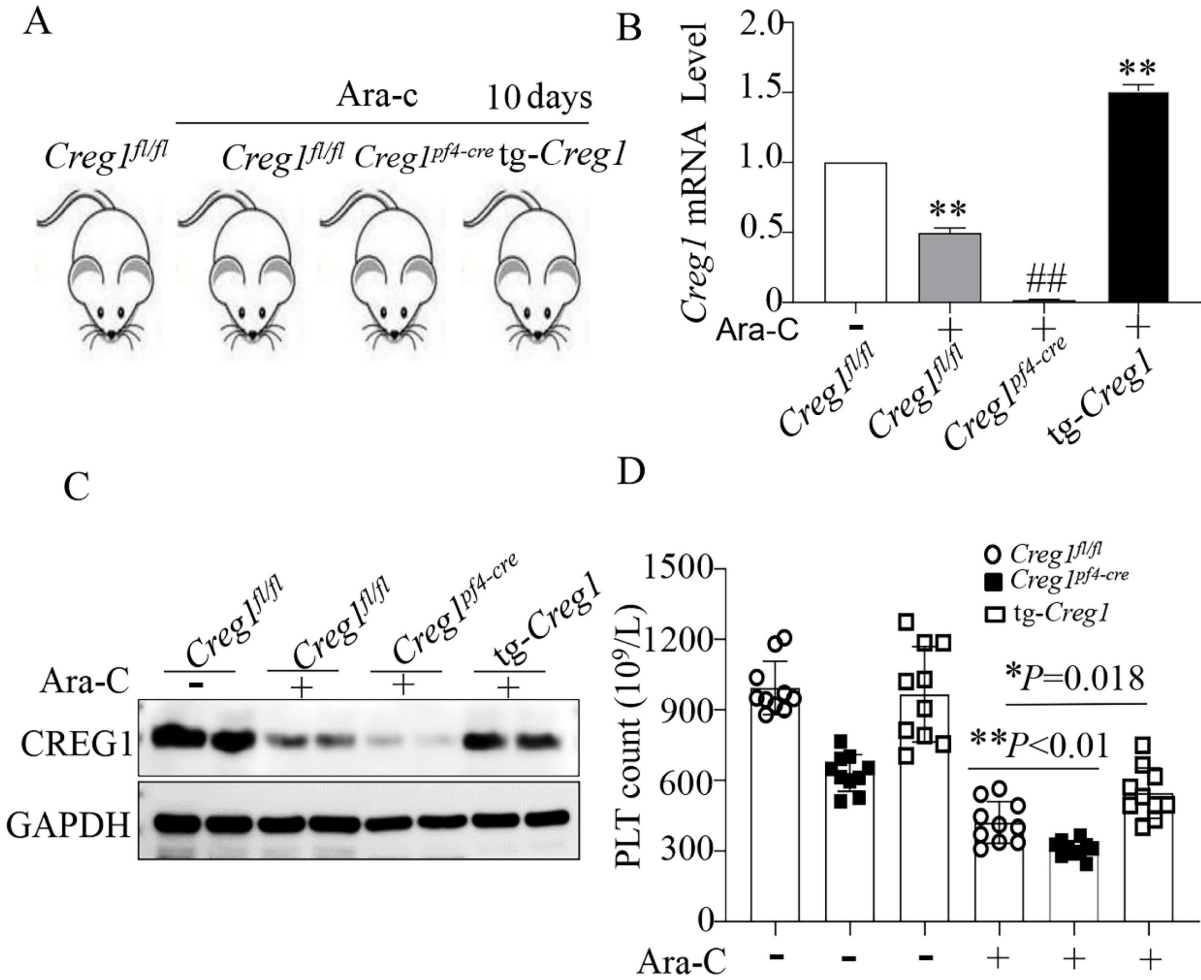
** The platelet count was increased in tg-*Creg1* mice.** (A) Mice treated intraperitoneally with Ara-c. (B) Expression of *Creg1* mRNA determined by real-time PCR. (C) Western blot was used to analyze CREG1 protein. (D) PLT count in the peripheral blood, (n=10). Values are means ± SEM. *P < 0.05, **P < 0.01 versus *Creg1^fl/fl^*.

We have shown the effects of CREG1 deficiency on the reduction of platelet production, but this is not sufficient to confirm the role of CREG1 in thrombocytopoiesis. Therefore, we performed rescue experiments using tg-*Creg1* mice (Supplementary Figures 2B-F) in a thrombocytopenia model treated with Ara-c* in vivo.* Under normal conditions, the platelet count in tg-*Creg1* mice was unchanged compared with that in the control (Figure 5D). When treated with Ara-c, we found that the expression of CREG1 mRNA and protein were decreased (Figures 5A-C). The *Creg1^fl/fl^* platelet count was markedly decreased; however, the platelet count of tg-*Creg1* mice was significantly increased compared with that in* Creg1^fl/fl^* mice treated with Ara-c (Figure 5D).

### Expression of CREG1 increased during PMA-induced Dami cell differentiation

Phorbol 12-myristate 13-acetate (PMA) has been widely used in megakaryocyte differentiation and polyploidy studies^22^. CD41 is a critical molecular marker protein for megakaryocyte maturation that increases with DNA ploidy during megakaryocyte differentiation. In this study, we found that the expression of CD41 at the mRNA and protein levels increased gradually from days 1 to 4 after PMA stimulation (Supplementary Figures 4A-C). RT-PCR and western blot analyses showed that the mRNA and protein expression of CREG1 increased with the time of PMA-induced differentiation (Supplementary Figures 4D-F). Immunofluorescence staining also confirmed that the expression of CREG1 increased significantly in Dami cells after PMA induction, and CREG1 was localized to the cytoplasm and around the nucleus (Supplementary Figure 4G).

### CREG1 knockdown prevented PMA-induced Dami cell differentiation and results in low ploidy

**Figure 6:**
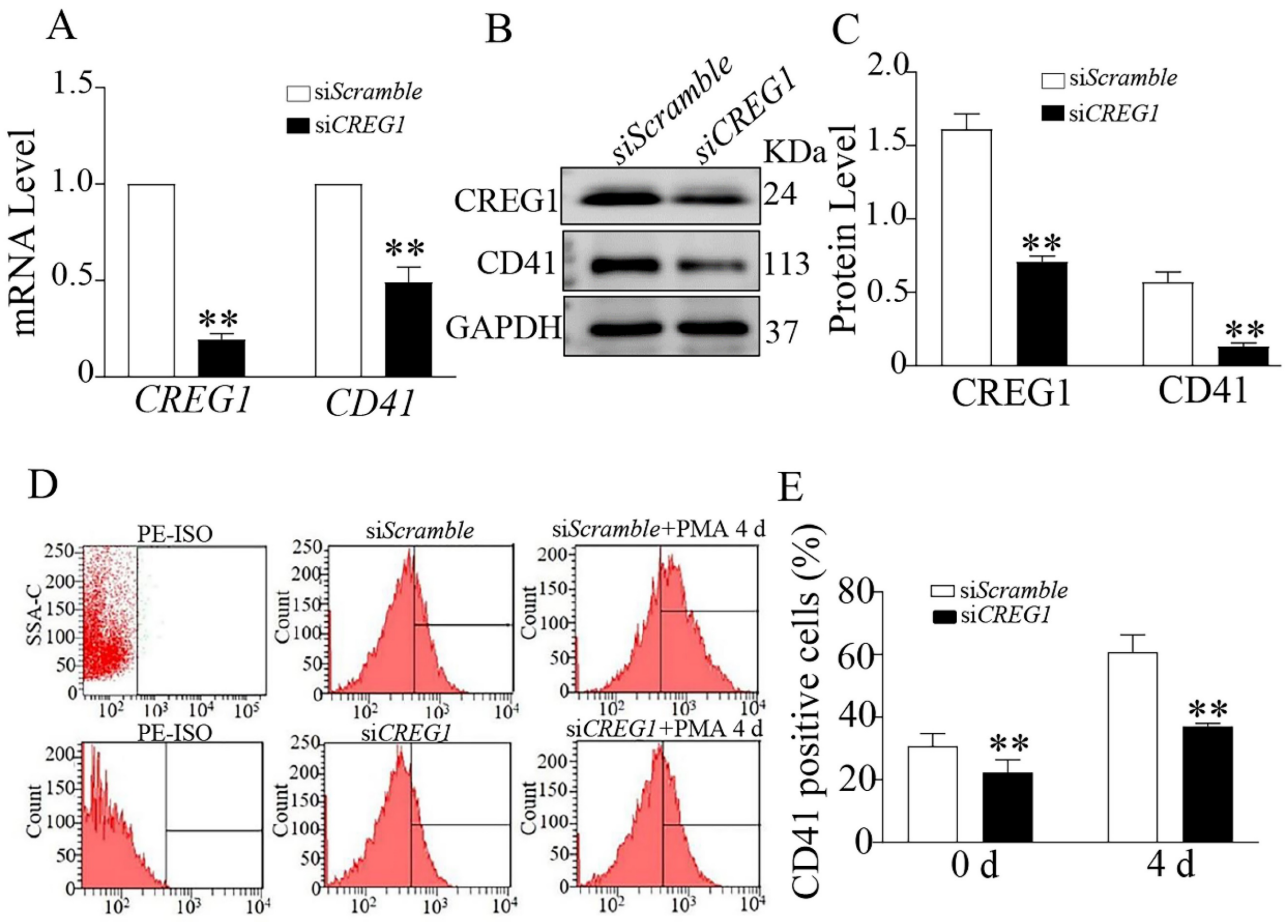
** Silencing of *CREG1* inhibited Dami cell differentiation.** (A) Real-time PCR analysis of *CREG1* and *CD41* mRNA expression. (B-C) Western blot was used to analyze and quantify the protein expression of CREG1 and CD41. (D-E) Flow cytometric analysis of si*Scramble*- and si*CREG1*-transfected Dami cells labeled with anti-CD41. Values are means ± SEM, n=3. **P < 0.01 versus si*Scramble*.

To determine whether CREG1 mediated PMA-induced Dami cell differentiation, we established a low-expression CREG1 cell model using *CREG1* siRNA. In the absence of PMA stimulation, CD41 expression was downregulated at both the mRNA and protein levels when *CREG1* siRNA was silenced (Figures 6A-C). Flow cytometry was used to investigate the expression of CD41 after PMA stimulation for four days with or without *CREG1* siRNA interference. The results revealed that the expression of CD41 increased gradually after 4 days of PMA stimulation, while it decreased significantly after *CREG1* siRNA interference compared to the control group (Figures 6D-E).

**Figure 7:**
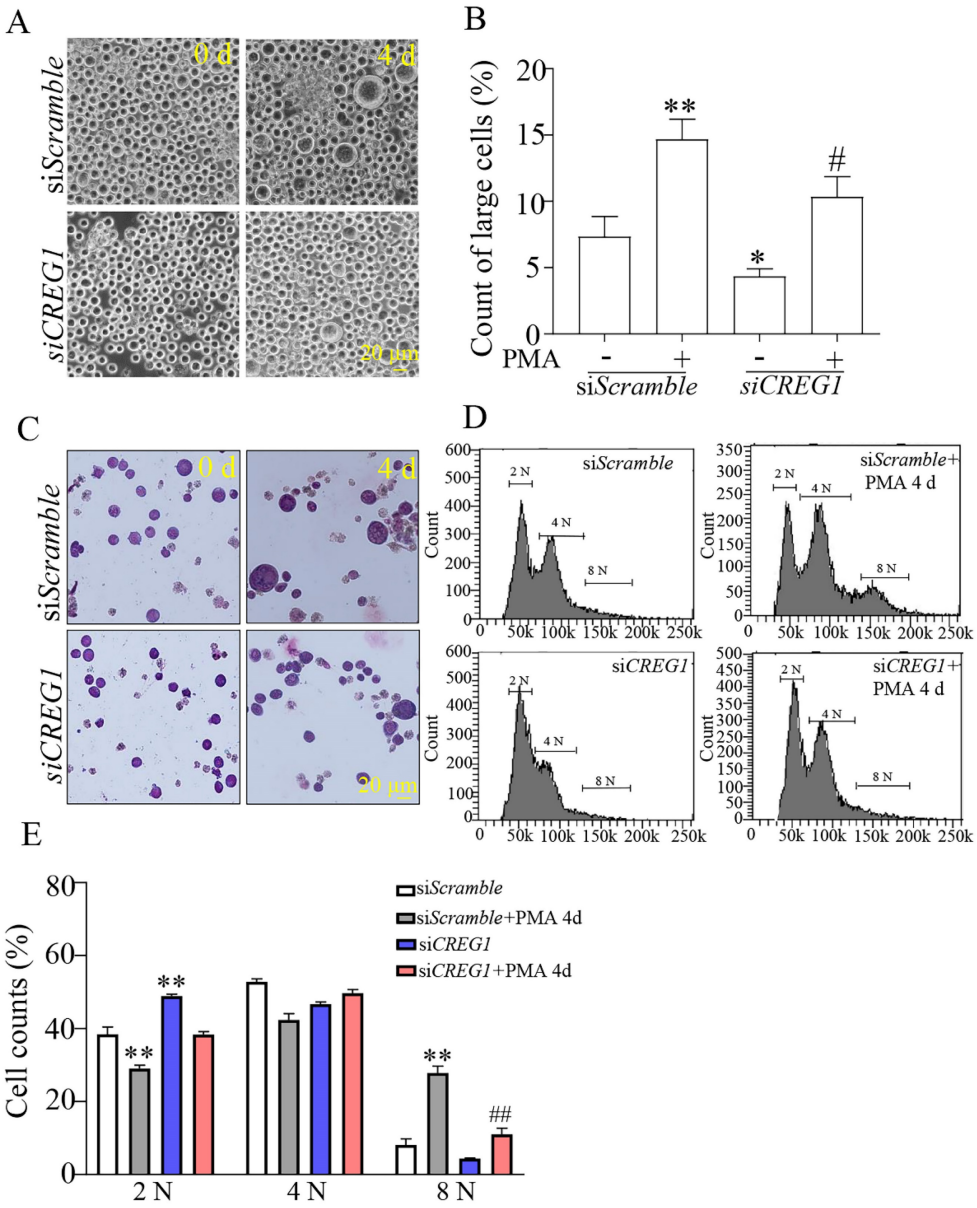
** Silencing of *CREG1* altered the morphology and caused low-ploidy developmental delay of megakaryocytes.** (A-B) The volume and cytoplasm of Dami cells decreased by *CREG1* knockdown, and the percentage of cells with diameters > 20 μm in three randomly selected fields was calculated. (C) Wright-Giemsa staining confirmed that knockdown of *CREG1* inhibited Dami cell differentiation and decreased the cytoplasmic volume and DNA ploidy. (D-E) Polyploidy analyzed by flow cytometry. Values are means ± SEM, n=3. *P < 0.05, **P < 0.01 versus si*Scramble*; ^#^*P* < 0.05, ^##^*P* < 0.01 versus si*Scramble* + PMA 4 d.

Dami cells are usually 10-15 μm in diameter, and it has been shown that the diameter of Dami cells differentiating into megakaryocytes is greater than 20 μm^23^. In our study, the proportion of Dami cells with a diameter < 20 μm in the* siCREG1* group was still higher than that in the control group. Similarly, on day 4 of PMA-induced stimulation, the proportion of Dami cells with a diameter > 20 μm in the *siCREG1* group was lower than that in the control group, which was consistent with the results of Giemsa staining (Figures 7A-C). DNA staining was performed using propidium iodide (PI) to distinguish between typical low ploidy (2-4 N) and high ploidy (≥8 N) in Dami cells. Dami cells exhibited polyploidy after 4 days of PMA stimulation, reaching 8 N or higher nearly 28% of the cell population. However, Dami cells in the si*CREG1* group reached 8 N or higher in only 11% of the cell population after four days of PMA stimulation (Figure 7D-E).

### The lack of CREG1 resulted in ERK1/2 pathway and PAK/LIMK1/Cofilin pathway inactivation *in vivo* and *in vitro*

**Figure 8:**
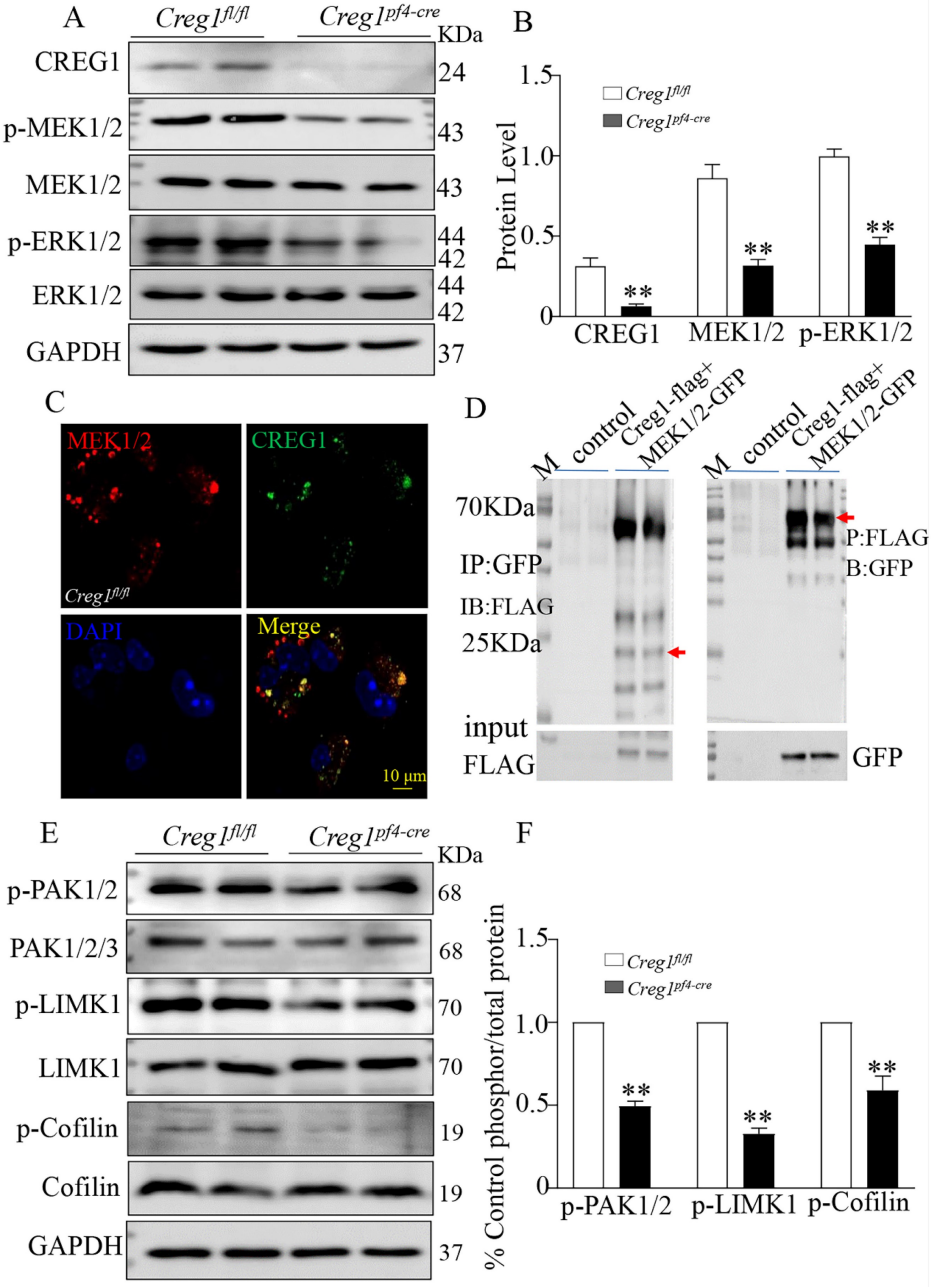
** Alteration of the ERK1/2 and PAK/LIMK1/Cofilin phosphorylation signaling pathways in *Creg1^pf4-cre^* megakaryocytes.** (A-B) Expression of CREG1, p-MEK1/2, and p-ERK1/2 was assayed by western blot in *Creg1^pf4-cre^* megakaryocytes. (C) Immunofluorescence staining of MEK1/2 and CREG1 in *Creg1^fl/fl^* megakaryocytes. (D) Co-immunoprecipitation of MEK1 and CREG1 in *Creg1^fl/fl^* megakaryocytes. (E-F) Expression of p-PAK1, p-LIMK1, and p-Cofilin1 was determined by western blot in *Creg1^pf4-cre^* megakaryocytes. Values are means ± SEM, n=3. **P < 0.01 versus *Creg1^fl/fl^*.

The ERK1/2 signaling pathway plays a critical role in regulating megakaryocyte differentiation and platelet formation^5^,^6^. The expression of ERK1/2 signaling pathway proteins was assayed in *Creg1^fl/fl^* and *Creg1^pf4-cre^* megakaryocytes cultured from BM. The results show that phosphorylated mitogen-activated protein kinase (P-MEK1/2) and phosphorylated extracellular signal-regulated kinase (p-ERK1/2) protein levels in *Creg1^pf4-cre^* megakaryocytes were significantly decreased (Figures 8A-B), but p-p38 and p-JNK expression levels did not change (Supplementary Figure 6A). Further experiments revealed that CREG1 interacted directly with MEK1/2 in 293T cells (Supplementary Figure 5) and megakaryocytes of* Creg1^fl/fl^* mice (Figure 8C), based on immunofluorescence staining and co-immunoprecipitation (Figure 8D). In Dami cells, loss of CREG1 significantly inhibited ERK1/2 pathway activity (Supplementary Figures 6B-C). The selective non-competitive MEK inhibitor U0126 has become an important pharmacological tool for studying the MEK-ERK1/2 signaling pathway^24^. It inhibits MEK activation and decreases ERK1/2 phosphorylation. In this study, treatment of Dami cells with U0126 did not affect the expression of CREG1, whereas the expression of p-ERK1/2 and CD41 were significantly reduced (Supplementary Figures 6D-E). We also found that the expression of p-ERK1/2 in megakaryocytes cultured from the BM of tg-*Creg1* mice was rescued compared with those of *Creg1^fl/fl^* mice when treated with cytosine arabinoside (Supplementary Figures 7A-B).

The PAK/LIMK1/Cofilin pathway is required for the regulation of megakaryocyte cytoskeletal protein organisation, polyploidy, and DMS polarization^3^. Studies have found that PAK (p21-activated kinase) gene knockout results in limited intracellular division during megakaryocyte formation, severe defects in megakaryocyte F-actin cytoskeleton dynamics, impaired phosphorylation of the PAK substrate LIM domain kinase 1 (LIMK1), and inhibition of platelet generation^25^. In this study, western blot analysis revealed that phosphorylation of PAK1 and LIMK1 was impaired in megakaryocytes cultured from the BM of *Creg1^pf4-cre^* mice, leading to a significant decrease in phosphorylated cofilin (Figures 8E-F). Cofilin is active in its non-phosphorylated state, and its ability to depolymerize F-actin is induced by LIMK1 phosphorylation, suggesting that *Creg1^pf4-cre^* megakaryocytes undergo more frequent F-actin fragmentation and depolymerization.

## Discussion

Thrombocytopenia is a common clinical condition caused mainly by abnormal megakaryocyte development and maturation. However, the exact signaling pathways involved in thrombocytopenia remain unclear. In this study, we identified CREG1 as an unknown regulator of megakaryocytes. We found that CREG1 deletion in megakaryocytes/platelets leads to a reduced platelet count with compensatory hyperplasia of megakaryocytes. CREG1 deficiency impaired megakaryocyte differentiation and PPF formation. Mechanistically, the lack of CREG1 resulted in inactivation of the ERK1/2 pathway (Supplementary Figure 8).

Many studies have demonstrated that CREG1 is an important glycoprotein that promotes the differentiation of myocardial cells, smooth muscle cells, and other cells^8^-^11^. However, whether CREG1 is expressed in megakaryocytes and whether it plays a role in promoting differentiation remains unknown. In our study, we found decreased expression of CREG1 in platelets from patients with thrombocytopenia. Furthermore, we showed that the expression of CREG1 was markedly downregulated at the mRNA and protein levels in megakaryocytes of mice with Ara-c induced thrombocytopenia. These results suggest that decreased CREG1 correlates with a decrease in the platelet count, but the underlying mechanism needs to be elucidated in future studies.

The main pathological causes of thrombocytopenia include a reduction in platelet production, platelet destruction, and excessive consumption^26^. We demonstrated that CREG1 deficiency impaired platelet production, along with decreased platelet counts and a compensatory upregulation of megakaryopoiesis. Megakaryocytes are derived from hematopoietic stem cells through hierarchical differentiation, after the hematopoietic stem cells are differentiated into megakaryocytic progenitor cells, they eventually form megakaryocytes through proliferation and terminal differentiation^27^,^28^. Previous studies have reported that CREG1 can induce smooth muscle, cardiomyogenic, and endothelial differentiation from embryonic stem cells. We speculated that CREG1 may be involved in the early proliferation and differentiation of megakaryocytic progenitor cells; however, we did not find evidence of this, which is a limitation of the present study. As CREG1 plays important roles in promoting differentiation, inhibition of apoptosis, and cell survival^10^, it was hypothesized that CREG1 regulates platelet apoptosis. However, we did not detect a significant effect on the expression of cleaved caspase-3 or annexin-V binding in *Creg1^fl/fl^* and *Creg1^pf4-cre^* platelets, suggesting that CREG1 was dispensable for apoptotic pathways in platelets.

Megakaryocytes undergo cytoskeletal alterations, which are pivotal for polyploidy and proplatelet formation during maturation. Studies have revealed that actin polymerization and filament turnover are critical for maintaining megakaryocyte cytoskeleton reorganization during DMS formation and megakaryocyte polarization^3^-^5^. In the present study, the lack of CREG1 led to marked disruption of filamentous actin in *Creg1^pf4-cre^* megakaryocytes. Moreover, DMS organization depends on F-actin dynamics to polarize megakaryocyte nuclei and membranes during platelet formation. We found that the DMS structure was disrupted in *Creg1^pf4-cre^* megakaryocytes, which could result in insufficient proplatelet formation. As expected, *Creg1^pf4-cre^* fetal liver-derived megakaryocytes produced fewer proplatelets. Impaired phosphorylation of PAK, its substrate LIMK, and cofilin is associated with DMS disruption^3^. The lack of CREG1 abrogated the activity of the PAK/LIMK1/Cofilin pathway, although the specific mechanisms were not elucidated in the present study. This may be the cause of reduced platelet production in *Creg1^pf4-cre^* mice.

Megakaryocytes polyploids are important for efficient platelet production, and polyploidization increases the volume and size of megakaryocytes. As megakaryocyte undergo maturation, they undergo endomitosis, which leads to polyploidy due to failure of karyokinesis and cytokinesis^29^-^30^. We found that *Creg1^pf4-cre^* megakaryocytes were arrested in the hypoploidy phase (2N-4N), but fewer cells were arrested in the high-ploidy phases (8N-32N), and deficiency of CREG1 prevented polyploidization *in vivo*. In the Dami cell line, CREG1 deletion also impaired cell differentiation, affected morphology, and caused low-ploidy developmental delay *in vitro*. Next, we explored the mechanism by which CREG1 deficiency caused polyploidy disorders. TPO is a polyploidization factor and has broader effects on hematopoietic stem cell and megakaryocyte differentiation^31^, CREG1 ablation did attenuate the effect TPO signaling pathway. The MAPK/ERK1/2 pathway is also important for megakaryocyte polyploidization. Many studies have shown that inhibition of the MAPK/ERK1/2 pathway prevents megakaryocyte polyploidization and differentiation in murine primary cells and cell lines^5^,^6^,^31^. Deficiency of CREG1 in megakaryocytes resulted in inactivation of the ERK1/2 pathway* in vivo* and *in vitro*; however, CREG1 overexpression in megakaryocytes from tg-*Creg1* mice enhanced p-ERK1/2 expression and rescued the platelet count compared to in the* Creg1^fl/fl^* mice, indicating that CREG1 regulates the ERK1/2 pathway to promote megakaryocyte polyploidization and differentiation.

## Conclusion

In summary, CREG1 may be involved in megakaryocyte differentiation and platelet production as a new regulatory factor that activates ERK1/2 signaling to promote megakaryocyte differentiation and platelet production. By identifying CREG1 as an important target, we have provided a theoretical basis and new ideas for the prevention and treatment of thrombocytopenia.

## Supplementary Material

Supplementary figures and table.
