# Supplementary material for: The role of CREG1 in megakaryocyte maturation and thrombocytopoiesis
Source: Int J Biol Sci. 2023 Jul 9;19(11):3614–27. doi: 10.7150/ijbs.78660 (PMC10367557; doi:10.7150/ijbs.78660)
Supplement: Supplementary file 1 — Supplementary figures and table. [file ijbsv19p3614s1.pdf]

Supplemental figures

Table 1 The study population baseline data

	total（n=20）	control group	patients	T/χ^2	P
age（years，$\bar{x}$±S）	60.05±6.37	59.70±6.84	60.40±6.20	0.240	0.813
Sex [n(%)]					
male	12(60.0)	6(60.0)	6(60.0)	0.000	1.000
female	8(40.0)	4(40.0)	4(40.0)		
BMI (kg/m^2)	23.90±2.37	23.80±2.00	24.00±2.80	0.184	0.856
Hypertension [n(%)]					
yes	7（35.0）	3(30.0)	4(40.0)	0.220	0.639
no	13（65.0）	7(70.0)	6(60.0)		
Diabetes [n(%)]					
yes	4(20.0)	1(10.0)	3(30.0)	0.313	0.576
no	16(80.0)	9(90.0)	7(70.0)		
Smoke [n(%)]					
yes	8(40.0)	4(40.0)	4(40.0)	0.000	1.000
no	12(60.0)	6(60.0)	6(60.0)		
Systolic pressure (mmHg，$\bar{x}$±S)	128.5±11.74	129.30±11.30	127.70±12.76	-0.297	0.770
diastolic pressure (mmHg，$\bar{x}$±S)	70.45±18.37	63.50±23.05	77.40±8.59	1.787	0.091
WBC(10^9/L)	6.37±1.99	6.16±2.00	6.59±2.07	0.472	0.643
RBC(10^{12}/L)	4.29±0.58	4.46±0.51	4.13±0.62	-1.312	0.206
PLT(10^9/L)	174.05±90.66	254.90±47.63	93.2±23.65	-9.615	<0.001
ALT(u/L)	21.90±9.92	23.37±8.86	20.44±11.16	-0.652	0.523
CKMB(u/L)	10.65±4.38	9.70±4.02	11.60±4.71	0.968	0.346
TG(mmol/L)	1.68±2.65	1.25±0.66	2.11±3.74	0.713	0.485
LDL-C(mmol/L)	2.16±0.84	2.34±0.93	1.98±0.75	-0.938	0.361
CHOL(mmol/L)	4.23±1.20	4.28±1.38	4.18±1.05	-0.176	0.863
CREA(umol/L)	65.66±11.01	65.98±9.14	65.35±13.13	-0.126	0.901

Supplemental figure 1

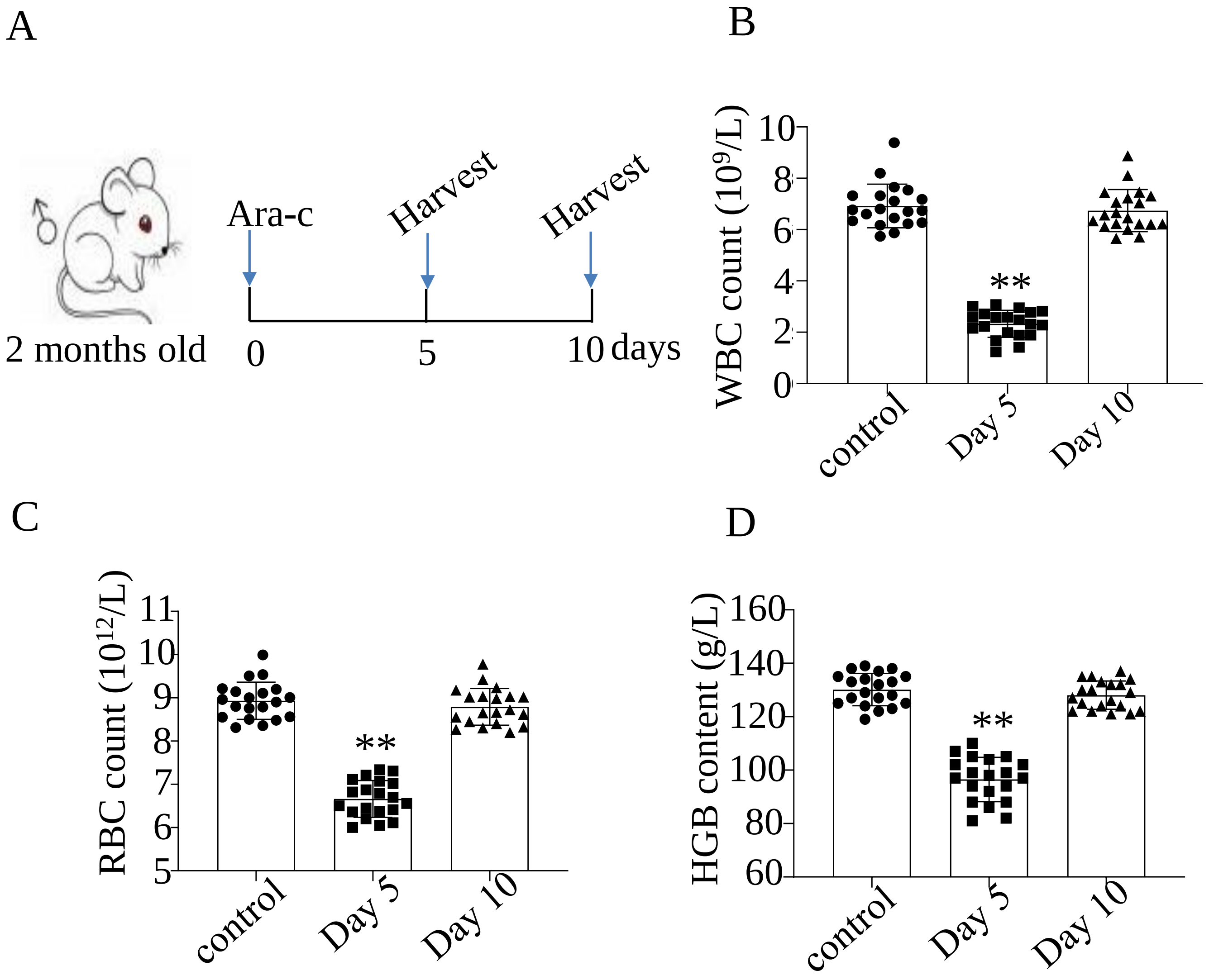

Supplemental Figure 1. WBC count, RBC count and HGB content in the peripheral blood of mice. (A) mice treated with Ara-c intraperitoneally. (B) WBC count. (C) RBC count. (D) HGB content. Values are presented as means ± SEM. n=20 mice/group. **P < 0.01 versus control. Ara-c: cytosine arabinoside.

Supplemental figure 2

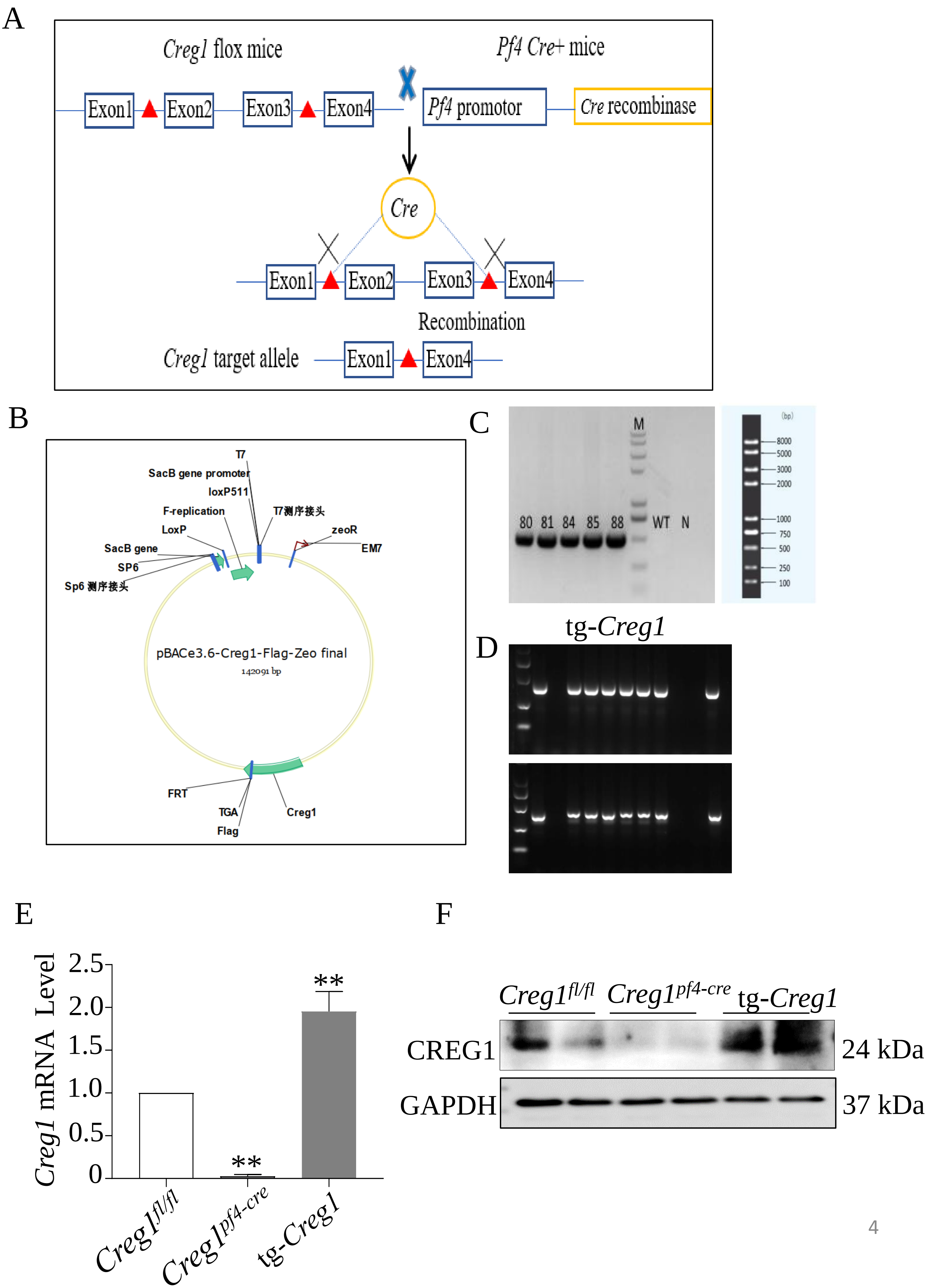

Supplemental Figure 2. (A-D) Generation of *Creg1*$^{pf4\text{-}cre}$ mice and tg-*Creg1* mice, and genotyped. (E-F) Quantitative real-time PCR (RT-PCR) and western blot confirmed the establishment of the model. Values are presented as means ± SEM, n=3. $^{**}P < 0.01$ versus *Creg1*$^{fl/fl}$.

Supplemental figure 3

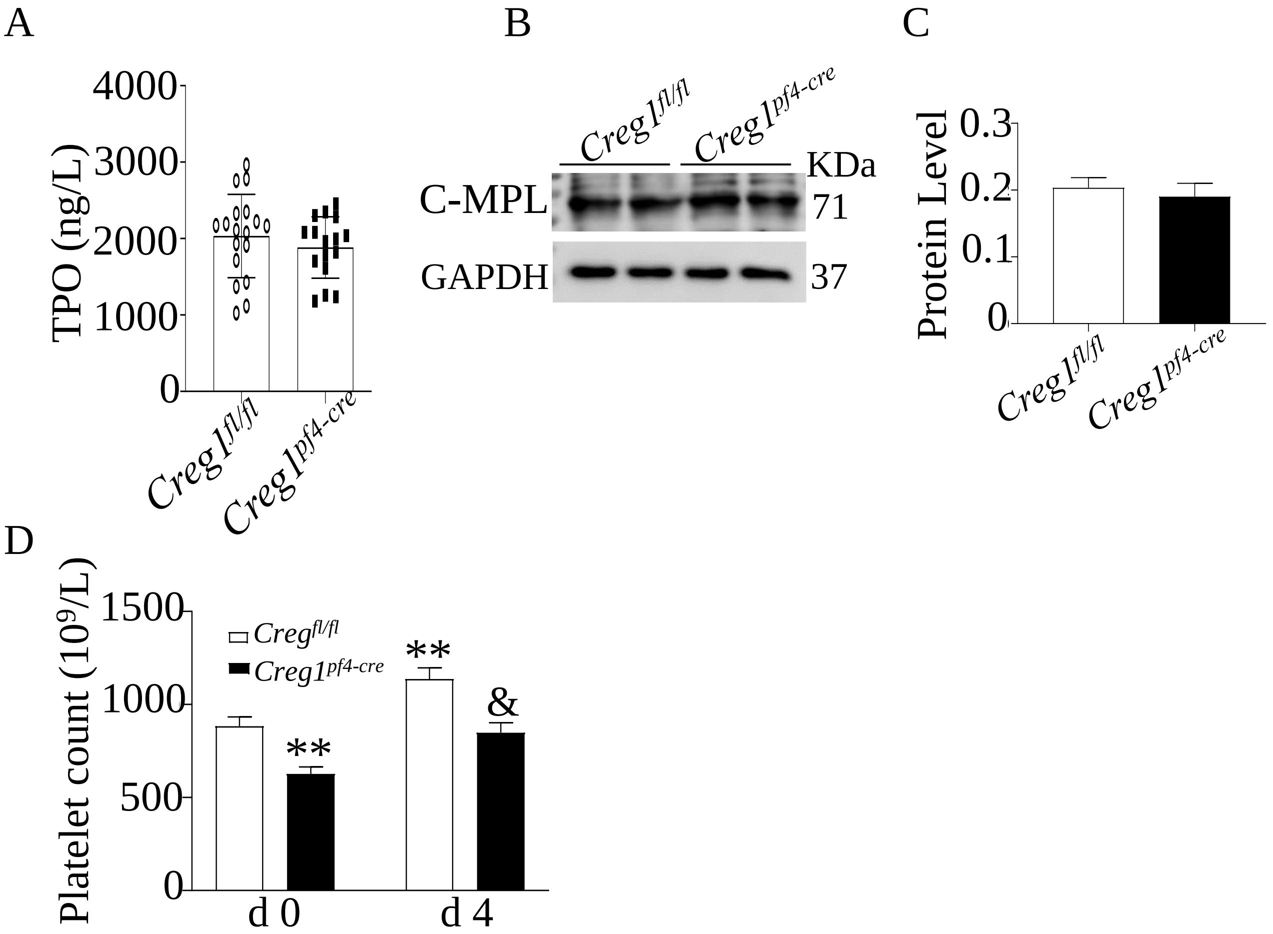

Supplemental Figure 3. The lack of CREG1 attenuated TPO signaling pathway. (A) TPO levels were determined in the serum of murine blood by using ELISA. (n=18). (B-C) Western blot was used to analyze C-MPL. (D) Platelet count after treatment with TPO (2 µg/animal per day). (n=3). Values are presented as means ± SEM. $^{**}P < 0.01$ versus *Creg*$^{fl/fl}$, $^{\&}P < 0.01$ versus *Creg1*$^{pf4\text{-}cre}$ d 0.

Supplemental figure 4

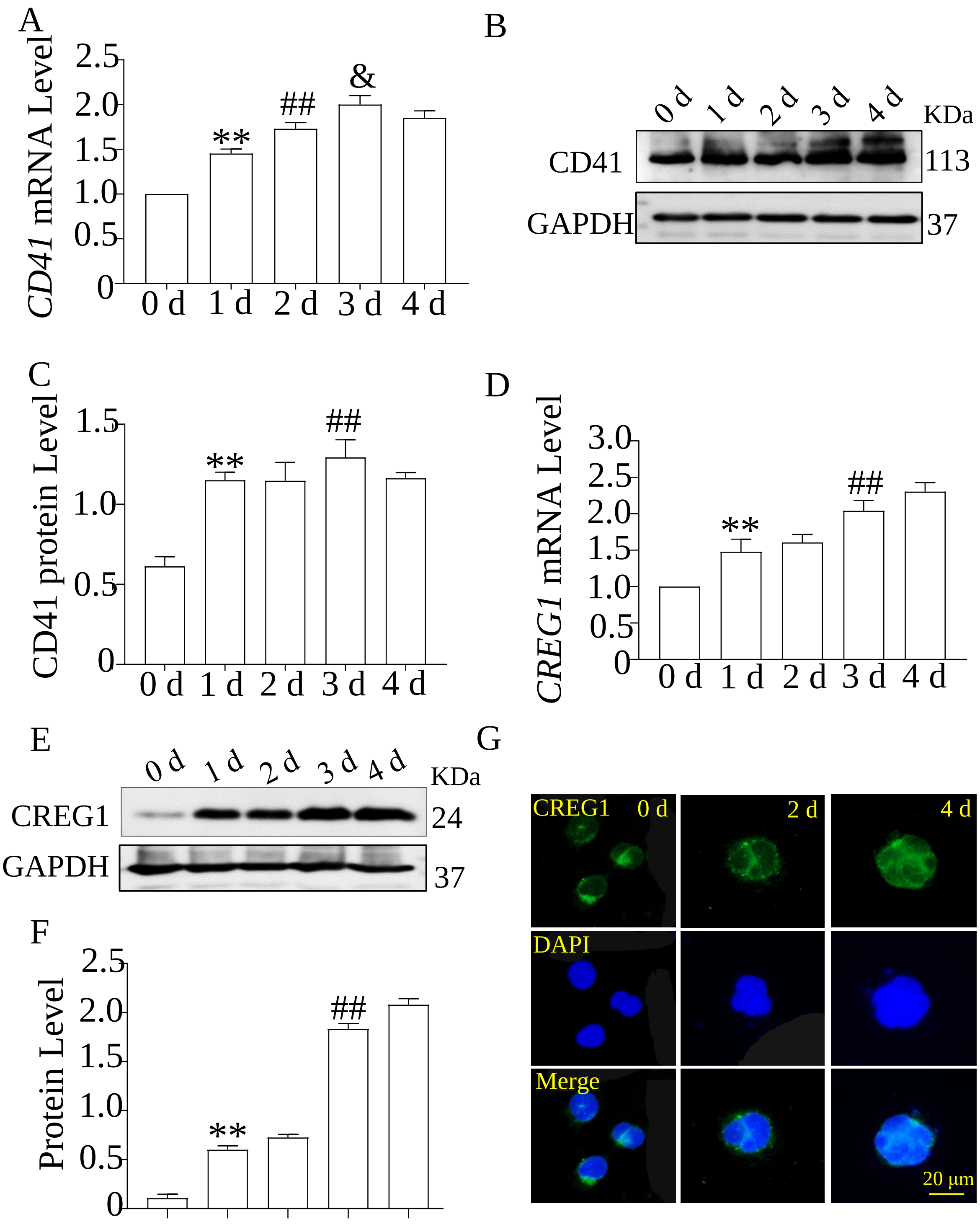

Supplemental figure 4. Expression of CREG1 in Dami cells was increased when stimulated by PMA. (A-C) Expression of CD41 was determined by real-time PCR and western blot after PMA treatment for 1 to 4 days. (n=3). (D-F) Expression of CREG1 was determined by real-time PCR and western blot after PMA treatment for 1 to 4 days. (n=3). (G) The localization and expression of CREG1 in Dami cells were determined by performing immunofluorescence staining. (n=5). Values are presented as means ± SEM. $^{**}P < 0.01$ versus control, $^{\#\#}P < 0.01$ versus 1 d, $^{\&}P < 0.01$ versus 2 d.

Supplemental figure 5

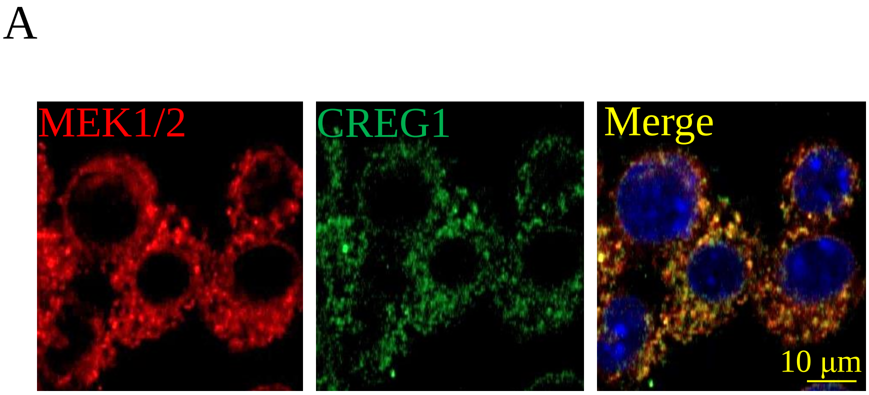

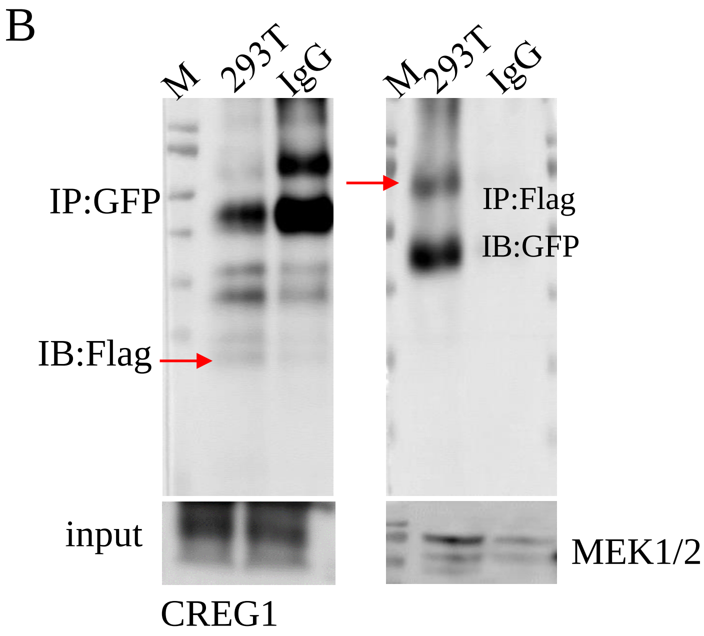

Supplemental figure 5. CREG1 directly combined with MEK1/2 in 293T cell. (A) Immunofluorescence staining of MEK1/2 and CREG1 in 293T cells. (B) Co-immunoprecipitation of MEK1 and CREG1 in 293T cells. (n=3).

Supplemental figure 6

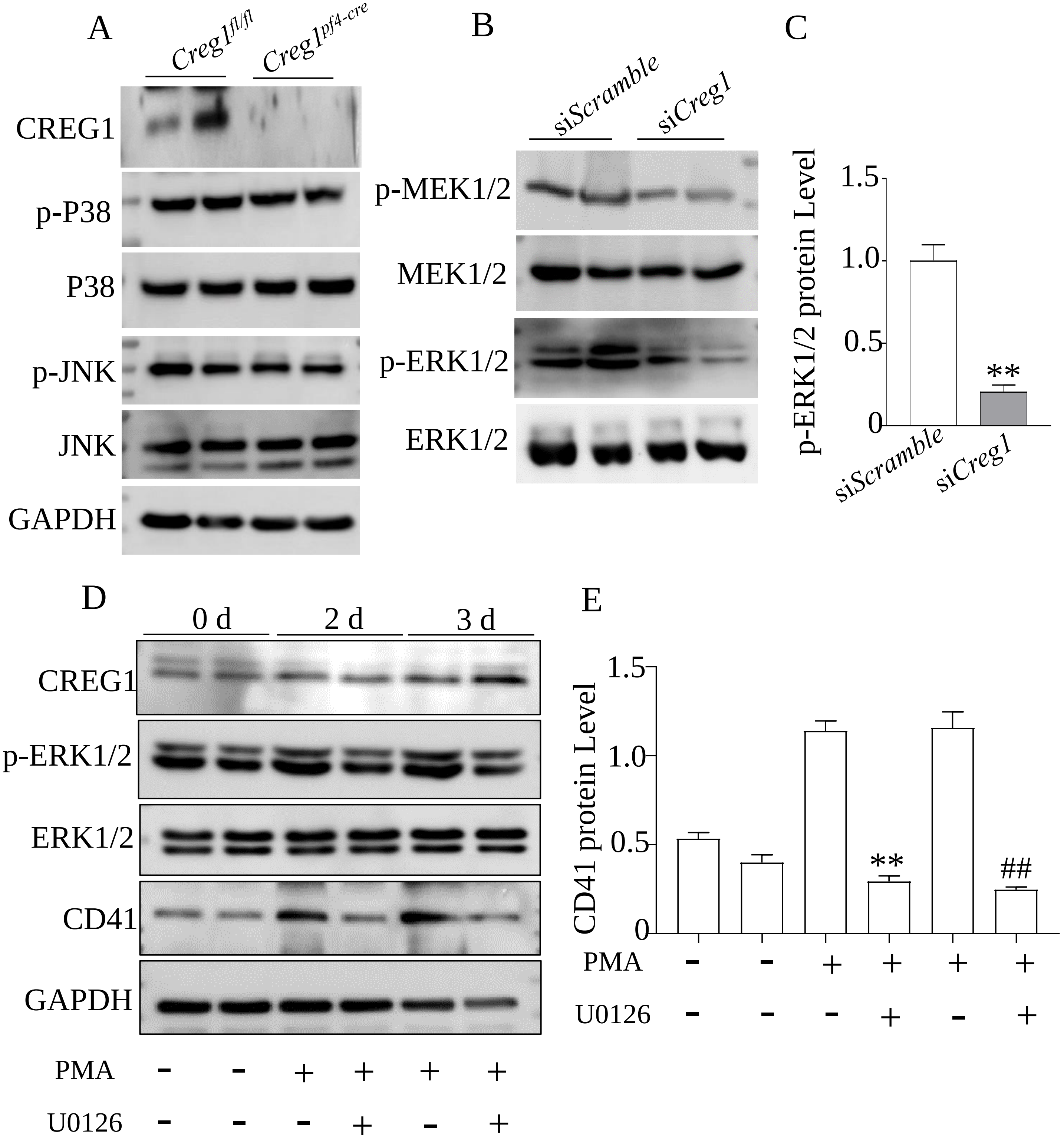

Supplemental figure 6. MEK1/2-ERK1/2 phosphorylation signaling pathways were abnormal when CREG1 silenced. (A) Expression of p-P38 and p-JNK was determined by western blot in *Creg1*$^{pf4\text{-}cre}$ mice (n=3). (B-C) Expression of p-MEK1/2 and p-ERK1/2 was determined by western blot in Dami cells (n=3). (D-E) Expression of CREG1, CD41 and p-ERK1/2 was detected by western blot (n=3). Values are presented as means ± SEM. $^{**}P < 0.01$ versus control or 2 d, $^{\#\#}P < 0.01$ versus 3 d.

Supplemental figure 7

A

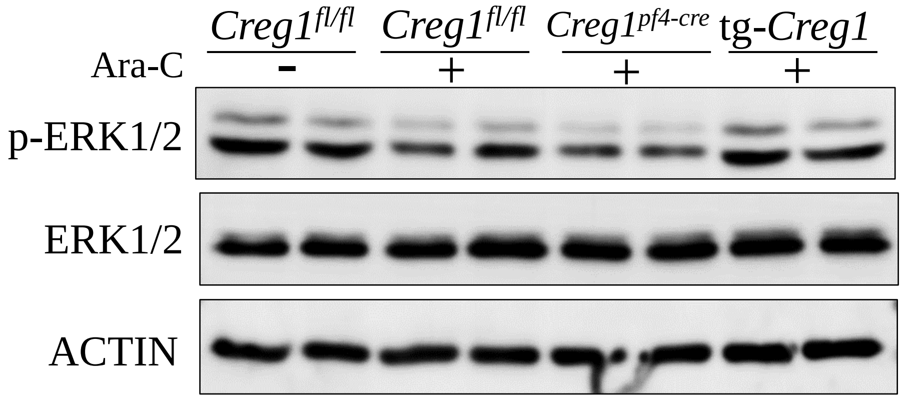

B

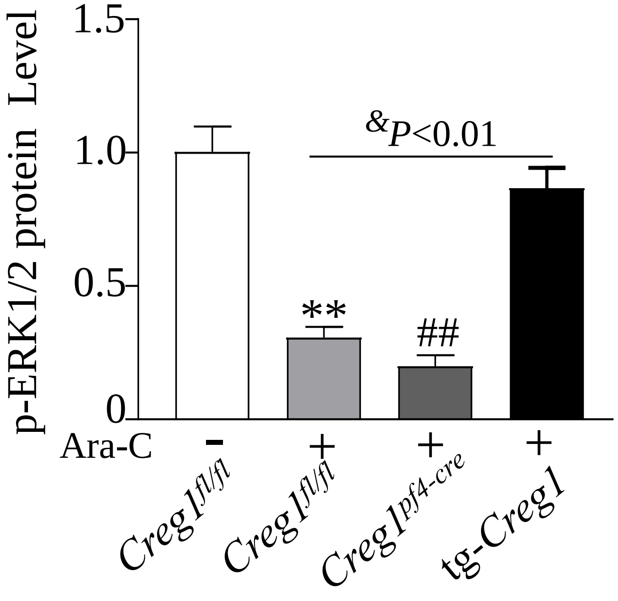

Supplemental figure 7. (A-B) Expression of p-ERK1/2 phosphorylation was determined by western blot (n=3). Values are presented as means ± SEM. ***P* < 0.01 versus *Creg1*$^{fl/fl}$, $^{\#\#}$*P* < 0.01 versus *Creg1*$^{fl/fl}$ +Ara-c, $^{\&}$*P* < 0.01 versus *Creg1*$^{fl/fl}$ +Ara-c.

Supplemental figure 8

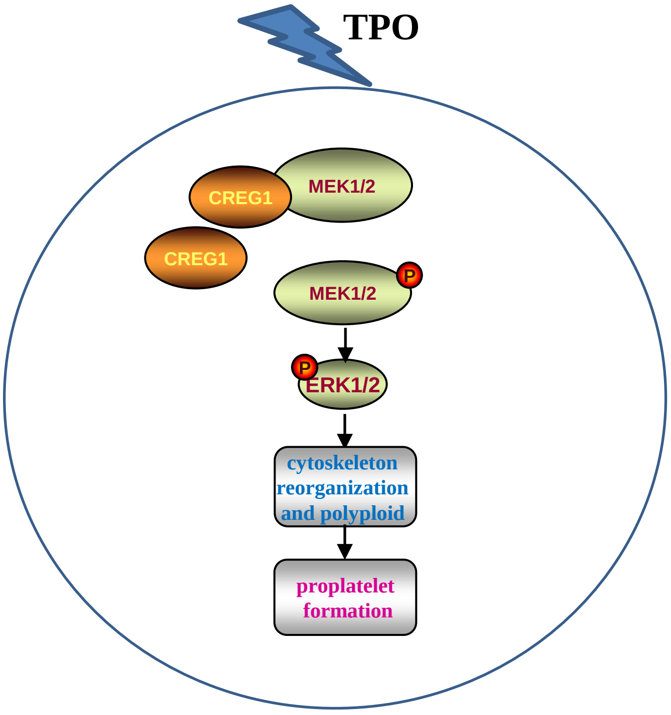

Supplemental Figure 8. A schematic picture was demonstrated how CREG1 regulated megakaryocytes differentiation and thrombopoiesis.
